# Supplementary material for: Simultaneous analytical method for 296 pesticide multiresidues in root and rhizome based herbal medicines with GC-MS/MS
Source: PLoS One. 2023 Jul 6;18(7):e0288198. doi: 10.1371/journal.pone.0288198 (PMC10325055; doi:10.1371/journal.pone.0288198)
Supplement: S1 Table — (PDF) [file pone.0288198.s001.pdf]

Supporting information

S1 Table. Retention times (t_R), multiple reaction monitoring (MRM) transitions, and collision energies (CEs) for 296 pesticides in GC-MS/MS.

No.	Pesticide name	t_R (min)	MRM transition Precursor ion > Product ion (CE, V)	
			Qualifier	Qualifier
1	2,6-Diisopropylnaphthalene	12.647	212.0>197.0(15)	197.0>167.0(30)
2	Acetochlor	9.460	223.0>146.0(10)	174.0>146.0(10)
3	EMA	8.070	135.0>120.0(10)	120.0>77.0(20)
4	HEMA	9.287	151.0>132.0(20)	151.0>117.0(40)
5	Acrinathrin	20.059	181.0>152.0(30)	181.0>127.0(30)
6	Alachlor	14.459	188.0>160.0(10)	160.0>130.0(30)
7	Aldrin	15.299	263.0>228.0(20)	263.0>193.0(40)
8	Dieldrin	16.993	263.0>228.0(25)	263.0>193.0(40)
9	Allidochlor	8.333	134.0>56.0(5)	132.0>56.0(10)
10	Ametryn	14.640	227.0>185.0(5)	227.0>170.0(10)
11	Anilofos	19.381	226.0>184.0(5)	226.0>157.0(15)
12-1	Aramite_1	17.041	187.0>65.0(15)	185.0>65.0(10)
12-2	Aramite_2	17.310	187.0>65.0(15)	185.0>65.0(10)
13	Aspon	15.147	211.0>115(10)	211.0>97.0(35)
14	Atrazine	12.853	215.0>200.0(10)	215.0>58.0(15)
15	Azaconazole	17.081	219.0>175.0(15)	217.0>173.0(15)
16	Benfluralin	11.679	292.0>264.0(10)	292.0>160.0(20)
17	Benfuresate	14.197	256.0>163.0(5)	163.0>121.0(5)
18	Benodanil	17.718	231.0>203.0(20)	231.0>76.0(25)
19	Benoxacor	13.959	176.0>120.0(5)	120.0>93.0(15)
20	Benzoylprop-ethyl	18.697	292.0>105.0(10)	105.0>77.0(10)
21	BHC-alpha	12.259	219.0>183.0(10)	219.0>145.0(20)
22	BHC-Beta	12.261	219.0>183.0(10)	219.0>145.0(20)
23	BHC-Delta	13.095	219.0>183.0(10)	219.0>145.0(20)
24	BHC-Gamma	13.079	219.0>183.0(10)	219.0>145.0(20)
25	Bifenox	19.405	341>310(10)	189.0>126.0(20)
26	Bifenthrin	18.998	181>166(15)	181.0>165.0(30)
27	Binapacryl	21.801	83 > 55 (10)	83 > 53 (15)
28	Boscalid	22.215	140.0>112(10)	140.0>76.0(25)
29	Bromobutide	14.341	232.0>176.0(10)	232.0>119.0(10)
30	Bromophos-ethyl	16.299	303.0>211.0(30)	97.0>65.0(20)
31	Bromophos-methyl	15.624	331.0>316.0(15)	125.0>47.0(10)
32	Bromopropylate	19.007	341.0>185.0(20)	341.0>155.0(40)
33	Bupirimate	17.000	273.0>193.0(10)	166.0>96.0(10)
34	Buprofezin	17.019	175.0>132.0(15)	172.0>57.0(15)
35	Butachlor	16.469	188.0>160.0(10)	176.0>147.0(10)
36	Butafenacil	21.467	331.0>180.0(20)	180.0>124.0(22)
37	Butralin	15.541	266.0>220.0(10)	266.0>174.0(20)
38	Butylate	9.354	156.0>57.0(5)	146.0>90.0(5)
39	Cadusafos	11.998	159.0>97.0(20)	158.0>97.0(20)

No.	Pesticide name	t_R (min)	MRM transition Precursor ion > Product ion (CE, V) Qualifier	Qualifier
40	Carbophenothion	17.967	342.0>157.0(15)	157.0>45.0(15)
41	Carboxin	17.042	235.0>143.0(10)	235.0>87.0(20)
42	Carfentrazone-ethyl	17.868	340.0>312.0(10)	312.0>151.0(20)
43	Chinomethionat	16.420	234.0>206.0(10)	234.0>148.0(30)
44	Chlorbenside	16.381	268.0>125.0(10)	143.0>108.0(10)
45	Chlorbufam	12.866	153.0>125.0(10)	153.0>90.0(20)
46-1	Chlordane_1	16.384	373.0>266.0(20)	272.0>237.0(20)
46-2	Chlordane_2	16.589	373.0>266.0(20)	272.0>237.0(20)
47	Chlorethoxyfos	11.070	153.0>125.0(5)	153.0>97.0(15)
48	Chlorfenapyr	17.257	247.0>227.0(15)	247.0>200.0(27)
49	Chlorfenson	16.735	175.0>111.0(10)	111.0>75.0(15)
50	Chlorflurenol-methyl	16.279	217.0>152.0(25)	215.0>152.0(20)
51	Chlornitrofen	17.938	317.0>287.0(15)	236.0>173.0(20)
52	Chlorobenzilate	17.403	251.0>111.0(20)	251.0>75.0(25)
53	Chloroneb	9.999	206.0>191.0(10)	191.0>113.0(15)
54	Chloropropylate	17.397	251.0>139.0(20)	139.0>111.0(15)
55	Chlorothalonil	13.433	266.0>231.0(20)	266.0>170.0(30)
56	Chlorpropham	11.594	213.0>171.0(10)	171.0>127.0(15)
57	Chlorpyrifos	15.242	314.0>258.0(20)	199.0>171.0(15)
58	Chlorpyrifos-methyl	14.675	286.0>271.0(25)	286.0>93.0(20)
59	Chlorthal-dimethyl	15.322	301.0>223.0(25)	299.0>221.0(25)
60	Chlorthion	15.492	297.0>109.0(15)	297.0>79.0(40)
61	Chlorthiophos	17.560	325.0>269.0(10)	297.0>269.0(5)
62	Chlozolinate	15.946	331.0>259.0(5)	259.0>188.0(10)
63	Cinidon-ethyl	25.304	358.0>330.0(10)	330.0>302.0(20)
64	Cinmethylin	14.698	169.0>107.0(10)	154.0>111.0(5)
65	Clomazone	12.949	204.0>107.0(25)	125.0>89.0(15)
66	Coumaphos	21.270	226.0>163.0(10)	210.0>182.0(5)
67	Cyanophos	13.189	243.0>109.0(10)	125.0>79.0(5)
68	Cyflufenamid	17.141	223.0>203.0(12)	188.0>88.0(40)
69	Cyfluthrin	21.927	226.0>206.0(10)	163.0>127.0(5)
70	Cyhalofop-buthyl	20.020	256.0>120.0(20)	229.0>109.0(15)
71	Cyhalothrin	20.155	208.0>181.0(5)	197.0>141.0(10)
72	Cypermethrin	22.245	165.0>91.0(10)	163.0>127.0(5)
73	Cyprazine	14.286	227.0>212.0(10)	212.0>109.0(30)
74	Cyprodinil	15.844	224.0>208.0(20)	224.0>77.0(40)
75	DDD (p,p)	17.545	237.0>165.0(25)	235.0>165.0(25)
76	DDE (p,p)	16.912	248.0>176.0(30)	246.0>176.0(30)
77	DDT (o,p)	17.558	237.0>165.0(25)	235.0>165.0(25)
78	DDT (p,p)	18.174	237.0>165.0(25)	235.0>165.0(25)
79	Deltamethrin	23.723	253.0>174.0(5)	181.0>152.0(30)
80	Tralomethrin	23.723	253.0>174.0(5)	181.0>152.0(30)
81	Desmetryn	14.201	213.0>171.0(5)	213.0>58.1(10)
82	Dialifos	20.646	357.0>97.0(25)	208.0>89.0(25)
83	Di-allate	12.071	234.0>192.0(15)	234.0>150.0(20)
84	Diazinon	131.313	199.0>135.0(10)	199.0>93.0(15)

No.	Pesticide name	t_R (min)	MRM transition Precursor ion > Product ion (CE, V) Qualifier	Qualifier
85	Dichlobenil	8.782	173.0>136.0(5)	171.0>136.0(25)
86	Dichlofenthion	14.222	279.0>223.0(10)	223.0>205.0(15)
87	Dichlofluanid	11.643	200.0>45.0(5)	200.0>44.0(25)
88	Dichlormid	8.829	172.0>108.0(5)	166.0>56.0(10)
89	Diclobutrazol	17.078	272.0>161.0(10)	270.0>159.0(15)
90	Diclofop-methyl	18.389	340.0>253.0(10)	281.0>120.0(15)
91	Dicloran	12.572	206.0>148.0(25)	206.0>124.0(30)
92	Dicofol	15.533	139.0>111.0(15)	139.0>75.0(35)
93	Dicrotophos	11.632	193.0>127.0(5)	127.0>95.0(15)
94	Diethatyl-ethyl	16.573	188.0>160.0(5)	162.0>147.0(10)
95	Diethofencarb	15.275	267.0>225.0(5)	225.0>168.0(10)
96-1	Difenoconazole_1	23.610	325.0>267.0(15)	325.0>265.0(15)
96-2	Difenoconazole_2	23.691	325.0>267.0(15)	325.0>265.0(15)
97	Diflufenican	18.392	266.0>218.0(25)	266.0>183.0(25)
98	Dimepiperate	16.179	145.0>112.0(5)	145.0>69.0(15)
99	Dimethachlor	14.224	197.0>148.0(10)	197.0>120(20)
100	Dimethametryn	15.933	212.0>122.0(5)	212.0>94.0(20)
101	Dimethenamid	14.226	230.0>154.0(10)	154.0>111.0(10)
102	Dimethipin	12.873	124.0>76.0(5)	118.0>58.0(5)
103-1	Dimethomorph_1	24.320	301.0>165.0(15)	301.0>152.0(15)
103-2	Dimethomorph_2	24.740	301.0>165.0(15)	301.0>152.0(15)
104-1	Dimethylvinphos_1	15.250	297.0>109.0(15)	295.0>109.0(15)
104-2	Dimethylvinphos_2	15.266	297.0>109.0(15)	295.0>109.0(15)
105	Diniconazole	17.482	268.0>232.0(15)	268.0>136.0(35)
106	Dinitramine	13.534	261.0>241.0(10)	261.0>195.0(20)
107	Dioxathion	13.062	125.0>97.0(10)	125.0>79.0(30)
108	Diphenamid	15.650	167.0>165.0(30)	167.0>152.0(20)
109	Diphenylamine	11.269	169.0>168.0(10)	169.0>167.0(30)
110	Dithiopyr	14.720	354.0>306.0(5)	354.0>286.0(15)
111	Edifenphos	17.993	201.0>109.0(15)	173.0>109.0(10)
112	Endosulfan-alpha	16.590	195.0>159.0(10)	195.0>125.0(20)
113	Endosulfan-beta	17.477	195.0>159.0(10)	195.0>125.0(30)
114	Endosulfan-sulfate	18.109	272.0>235.0(20)	272.0>117.0(40)
115	Endrin	16.997	263.0>228.0(20)	263.0>193.0(40)
116	Endrin-ketone	18.962	317.0>281.0(10)	317.0>245.0(20)
117	EPN	19.063	157.0>110.0(20)	157.0>77.0(20)
118	Epoxiconazole	18.646	192.0>138.0(15)	192.0>111.0(25)
119	EPTC	8.823	132.0>90.0(5)	128.0>86.0(5)
120-1	Etaconazole_1	17.429	245.0>173.0(20)	173.0>145.0(15)
120-2	Etaconazole_2	17.493	245.0>173.0(20)	173.0>145.0(15)
121	Ethalfluralin	11.405	316.0>276.0(5)	276.0>202.0(15)
122	Ethion	17.529	231.0>175.0(10)	231.0>129.0(25)
123	Ethofumesate	14.990	207.0>161.0(5)	207.0>137.0(10)
124	Ethoprophos	11.323	158.0>114.0(5)	158.0>97.0(20)
125	Ethychlozate	16.008	165.0>138.0(10)	165.0>102.0(15)
126	Etoxazole	19.182	300.0>270.0(25)	204.0>176.0(10)

No.	Pesticide name	t_R (min)	MRM transition Precursor ion > Product ion (CE, V)	
			Qualifier	Qualifier
127	Etridiazole	9.560	211.0>183.0(10)	211.0>140.0(25)
128	Fenamidone	19.266	238.0>103.0(20)	238.0>91.0(30)
129	Fenarimol	20.441	251.0>139.0(15)	219.0>107.0(10)
130	Fenbuconazole	21.733	198.0>129.0(5)	129.0>78.0(20)
131	Fenchlorphos	14.683	285.0>270.0(15)	285.0>93.0(25)
132	Fenclorim	12.288	224.0>189.0(20)	189.0>104.0(10)
133	Fenfuram	13.636	201.0>109.0(25)	109.0>53.0(20)
134	Fenitrothion	14.951	277.0>260.0(5)	277.0>109(20)
135	Fenobucarb	10.960	150.0>121.0(5)	121.0>103.0(15)
136	Fenothiocarb	16.511	160.0>106.0(10)	160.0>72.0(15)
137	Fenoxanil	17.260	189.0>154.0(10)	189.0>125.0(10)
138	Fenpropathrin	19.222	265.0>210.0(10)	265.0>89.0(40)
139	Fenpropimorph	15.373	128.0>110.0(10)	128.0>70.0(15)
140	Fenpyrazamine	20.444	230.0>188.0(10)	230.0>117.0(30)
141	Fenson	15.627	268.0>77.0(20)	141.0>77.0(10)
142	Fenthion	15.323	278.0>169.0(20)	278.0>109.0(20)
143-1	Fenvalerate_1	23.143	169.0>127.0(10)	167.0>125.0(10)
143-2	Fenvalerate_2	23.365	169.0>127.0(10)	167.0>125.0(10)
144	Fipronil	15.887	367.0>255.0(30)	367.0>213.0(30)
145	Flamprop-isopropyl	17.390	105.0>77.0(15)	105.0>51.0(40)
146	Fluacrypyrim	16.533	204.0>189.0(5)	145.0>102.0(30)
147	Fluazifop-butyl	17.257	282.0>238.0(25)	282.0>91.0(5)
148	Fluchloralin	13.342	326.0>63.0(15)	306.0>264.0(5)
149-1	Flucythrinate_1	22.318	199.0>107.0(25)	157.0>107.0(15)
149-2	Flucythrinate_2	22.541	199.0>107.0(25)	157.0>107.0(15)
150	Fluensulfone	10.882	119.0>92.0(10)	119.0>59.0(30)
151	Flufenpyr-ethyl	17.351	408.0>345.0(15)	321.0>286.0(15)
152	Flumetralin	16.451	143.0>117.0(20)	143.0>107.0(30)
153	Flumioxazine	22.640	354.0>326.0(10)	287.0>259.0(5)
154	Fluopyram	16.013	173.0>145.0(20)	173.0>95.0(35)
155	Flurochloridone	15.539	311.0>174.0(20)	187.0>109.0(20)
156	Fluorodifen	16.670	190.0>126.1(20)	190.0>75.0(20)
157	Fluquinconazole	21.286	340.0>286.0(30)	340.0>108.0(40)
158	Flurtamone	19.575	333.0>120.0(15)	199.0>157.0(20)
159	Flusilazole	16.996	233.0>165.0(20)	233.0>152.0(20)
160	Flutianil	22.676	231.0>216.0(5)	231.0>200.0(10)
161-1	Fluvalinate_1	23.262	250.0>200.0(20)	250.0>55.0(20)
161-2	Fluvalinate_2	23.329	250.0>200.0(20)	250.0>55.0(20)
162	Fluxapyroxad	18.971	159.0>139.0(10)	159.0>43.0(30)
163	Fonofos	13.285	246.0>137.0(5)	246.0>109.0(15)
164	Formothion	14.026	170.0>93.0(15)	126.0>93.0(5)
165	Fthalide	15.566	243.0>215.0(5)	241.0>213.0(10)
166	Halfenprox	22.213	265.0>117.0(10)	263.0>129.0(40)
167	Heptachlor	14.653	274.0>237.0(20)	272.0>237.0(20)
168	Heptachlor epoxide	15.995	353.0>253.0(30)	217.0>182.0(20)
169	Heptenophos	10.528	124.0>89.0(15)	124.0>63.0(40)

No.	Pesticide name	t_R (min)	MRM transition<br>Precursor ion > Product ion (CE, V)<br>Qualifier	Qualifier
170	Hexachlorbenzene	13.350	284.0>249.0(21)	284.0>214.0(30)
171	Hexythiazox	16.398	184.0>149.0(5)	156.0>155.0(5)
172	Indanofan	19.388	159.0>103.0(15)	139.0>75.0(35)
173	Indoxacarb	24.819	203.0>134.0(15)	203.0>106.0(15)
174	Ipconazole	20.302	125.0>99.0(20)	125.0>89.0(20)
175	Iprobenfos	13.905	204.0>121.0(35)	204.0>91.0(10)
176	Iprodione	18.839	314.0>245.0(10)	314.0>56.0(20)
177	Isazofos	13.597	257.0>162.0(5)	208.0>166.0(10)
178	Isofenphos	15.964	213.0>185.0(5)	213.0>121.0(20)
179	Isofenphos-methyl	15.730	199.0>121.0(15)	199.0>93.0(30)
180	Isoprocarb	10.253	136.0>121.0(10)	136.0>77.0(30)
181	Isopropalin	16.686	264.0>222.0(10)	238.0>165.0(10)
182	Isoprothiolane	16.790	189.0>145.0(10)	189.0>89.0(20)
183	Isopyrazam	20.635	359.0>159.0(40)	159.0>139.0(10)
184	Isotianil	18.105	297.0>180.0(15)	180.0>91.0(20)
185	Isoxadifen-ethyl	17.875	222.0>205.0(5)	222.0>204.0(20)
186	Kresoxim-methyl	17.009	131.0>89.0(35)	116.0>89.0(15)
187	Leptophos	19.817	377.0>362.0(25)	171.0>77.0(25)
188	Mefenpyr-diethyl	18.638	299.0>253.0(10)	253.0>190.0(20)
189	Mepanipyrim	16.588	223.0>222.0(10)	222.0>221.0(20)
190	Mepronil	17.741	269.0>119.0(15)	269.0>91.0(40)
191	Metalaxyl	14.631	249.0>190.0(5)	234.0>146.0(20)
192	Methidathion	16.332	145.0>85.0(5)	145.0>58.0(15)
193	Methoprotryne	17.071	256.0>212.0(15)	256.0>170.0(25)
194	Methoxychlor	19.189	227.0>169.0(25)	227.0>141.0(40)
195	Methyl trithion	17.407	157.0>121.0(35)	125.0>47.0(15)
196	Metolachlor	15.204	238.0>162.0(10)	162.0>133.0(15)
197	Metribuzin	14.353	198.0>110.0(10)	198.0>82.0(20)
198	MGK_264	15.888	164.0>98.0(10)	164.0>67.0(5)
199	Mirex	21.116	272.0>237.0(20)	272.0>143.0(10)
200	Molinate	10.380	187.0>126.0(5)	126.0>98.0(5)
201	Monolinuron	12.875	214.0>61.0(5)	126.0>99.0(15)
202	Myclobutanil	16.974	179.0>152.0(5)	179.0>125.0(15)
203	Nitrapyrin	9.544	196.0>135.0(20)	194.0>133.0(20)
204	Nitrothal-isopropyl	15.508	236.0>194.0(10)	194.0>120.0(20)
205-1	Nonachlor_1	16.630	409.0>300.0(15)	407.0>300.0(30)
205-2	Nonachlor_2	17.558	409.0>300.0(15)	407.0>300.0(30)
206	Nuarimol	20.443	139.0>111.0(20)	107.0>52.0(15)
207	O-Phenylphenol	10.178	169.0>141.0(15)	169.0>115.0(30)
208	Oxadiazon	16.899	258.0>175.0(15)	175.0>112.0(5)
209	Oxadixyl	17.507	163.0>132.0(5)	163.0>117.0(25)
210	Oxyfluorfen	16.979	361.0>300.0(20)	300.0>223.0(20)
211	Paclobutrazol	16.470	236.0>167.0(5)	236.0>125.0(10)
212	Parathion	17.920	291.0>109.0(10)	291.0>81.0(45)
213	Parathion-ethyl	15.363	291.0>109.0(10)	291.0>81.0(40)
214	Parathion-methyl	14.474	263.0>109.0(15)	263.0>79.0(35)

No.	Pesticide name	t_R (min)	MRM transition Precursor ion > Product ion (CE, V)	
			Qualifier	Qualifier
215	Penconazole	15.937	248.0>192.0(20)	248.0>157.0(30)
216	Pendimethalin	15.815	252.0>208.0(5)	252.0>162.0(10)
217	Penflufen	17.672	274.0>141.0(15)	141.0>84.0(15)
218	Pentachlorobenzonitrile	13.068	275.0>240.0(20)	275.0>179.0(30)
219	Penthiopyrad	17.469	302.0>177.0(20)	177.0>101.0(20)
220	Pentoxazone	19.732	287.0>70.0(10)	285.0>70.0(15)
221-1	Permethrin_1	21.124	183.0>168.0(20)	183.0>155.0(10)
221-2	Permethrin_2	21.275	183.0>168.0(20)	183.0>155.0(10)
222	Perthane	17.294	223.0>193.0(20)	223.0>165.0(20)
223	Phenthoate	16.082	274.0>125.0(15)	274.0>121.0(10)
224	Phosalone	19.793	182.0>138.0(5)	182.0>102.0(15)
225	Phosmet	19.023	160.0>133.0(10)	160.0>77.0(30)
226-1	Phosphamidon_1	13.782	264.0>127.0(10)	127.0>109.0(15)
226-2	Phosphamidon_2	14.137	264.0>127.0(10)	127.0>109.0(15)
227	Picoxystrobin	16.534	335.0>173.0(10)	145.0>102.0(30)
228	Piperonyl butoxide	18.400	176.0>145.0(10)	176.0>131.0(25)
229	Pirimicarb	13.864	238.0>166.0(10)	166.0>96.0(15)
230	Pirimiphos-ethyl	15.611	318.0>166.0(10)	304.0>168.0(10)
231	Pirimiphos-methyl	14.894	290.0>125.0(20)	233.0>151.0(5)
232	Pretilachlor	16.790	262.0>202.0(5)	162.0>132.0(20)
233	Prochloraz	21.343	308.0>70.0	180.0>138.0
234	2,4,6-Trichlorophenol	8.889	196.0>97.0(40)	132.0>97.0(15)
235	Procymidone	16.152	285.0>96.0(5)	283.0>96.0(5)
236	Prodiamine	14.933	321.0>279.0(10)	275.0>255.0(10)
237	Profenofos	16.565	339.0>269.0(10)	337.0>267.0(10)
238	Profluralin	13.051	318.0>199.0(20)	318.0>55.0(10)
239	Prohydrojasmon	13.586	184.0>83.0(15)	153.0>97.0(5)
240	Prometon	12.719	210.0>168.0(10)	183.0>168.0(5)
241	Prometryn	14.699	241.0>199.0(10)	226.0>184.0(10)
242	Propachlor	11.005	176.0>92.0(20)	169.0>93.0(10)
243	Propanil	14.271	217.0>161.0(25)	161.0>126.0(25)
244	Propazine	12.935	229.0>58.0(15)	214.0>172.0(10)
245	Propetamphos	13.139	194.0>166.0(5)	138.0>64.0(20)
246	Propham	9.601	179.0>93.0(15)	137.0>93.0(10)
247-1	Propiconazole_1	18.015	259.0>173.0(5)	259.0>69.0(10)
247-2	Propiconazole_2	18.119	259.0>173.0(5)	259.0>69.0(10)
248	Propisochlor	14.572	162.0>147.0(15)	162.0>120.0(20)
249	Propyzamide	13.267	173.0>145.0(20)	173.0>109.0(35)
250	Prothiofos	16.779	267.0>239.0(10)	267.0>221.0(20)
251	Pyracarbolid	15.654	217.0>125.0(15)	125.0>107.0(15)
252	Pyraclofos	20.742	194.0>139.0(10)	194.0>138.0(20)
253	Pyraflufen-ethyl	18.064	412.0>349.0(10)	349.0>307.0(15)
254	Pyrazophos	20.381	232.0>204.0(10)	221.0>193.0(10)
255	Pyridalyl	22.528	204.0>176.0(10)	204.0>148.0(20)
256	Pyrifenox	16.408	187.0>124.0(20)	171.0>100.0(25)
257	Pyriftalid	20.483	318.0>274.0(5)	318.0>273.0(20)

No.	Pesticide name	t_R (min)	MRM transition Precursor ion > Product ion (CE, V) Qualifier	Qualifier
258	Pyrimethanil	13.444	198.0>158.0(20)	198.0>118.0(35)
259-1	Pyriminobac-methyl_1	17.365	302.0>256.0(20)	302.0>230.0(15)
259-2	Pyriminobac-methyl_2	18.032	302.0>256.0(20)	302.0>230.0(15)
260	Quinalphos	16.104	146.0>118.0(10)	149.0>91.0(30)
261	Quinoxyfen	18.053	307.0>237.0(20)	237.0>208.0(30)
262	Quintozene	12.977	295.0>237.0(20)	249.0>214.0(10)
263	Quizalofop-ethyl	22.344	372.0>299.0(10)	299.0>255.0(15)
264	Silafluofen	22.662	286.0>258.0(10)	179.0>91.0(10)
265	Simeconazole	14.485	121.0>101.0(15)	121.0>75.0(35)
266	Simetryn	14.569	213.0>185.0(10)	213.0>170.0(10)
267	Spiromesifen	18.684	272.0>254.0(10)	272.0>209.0(10)
268-1	Spiroxamine_1	14.443	198.0>126.0(5)	100.0>72.0(5)
268-2	Spiroxamine_2	14.944	198.0>126.0(5)	100.0>72.0(5)
269	Sulfotep	11.766	322.0>146.0(25)	202.0>146.0(10)
270	Tebuconazole	17.880	252.0>127.0(30)	250.0>125.0(30)
271	Tebufenpyrad	19.349	333.0>171.0(20)	276.0>171.0(10)
272	Tebupirimfos	13.851	276.0>234.0(5)	261.0>137.0(15)
273	Tecnazene	10.860	215.0>179.0(25)	213.0>142.0(25)
274	Tefluthrin	13.623	177.0>137.0(20)	177.0>127.0(20)
275	Terbacil	13.596	161.0>88.0(20)	160.0>76.0(15)
276	Terbumeton	12.977	210.0>100.0(20)	169.0>154.0(10)
277	Terbutryn	14.949	241.0>185.0(5)	185.0>170.0(5)
278	Tetrachlorvinphos	16.420	331.0>109.0(25)	329.0>109.0(25)
279	Tetraconazole	15.433	336.0>218.0(20)	336.0>204.0(40)
280	Tetradifon	19.667	229.0>201.0(10)	159.0>111.0(20)
281-1	Tetramethrin_1	18.899	164.0>107.0(15)	164.0>77.0(25)
281-2	Tetramethrin_2	19.074	164.0>107.0(15)	164.0>77.0(25)
282	Tetrasul	17.752	323.0>251.0(15)	321.0>252.0(15)
283	Thifluzamide	16.915	194.0>166.0(10)	194.0>125.0(25)
284	Thiometon	12.426	125.0>47.0(25)	93.0>63.0(5)
285	Thionazin	10.937	248.0>140.0(10)	192.0>96.0(15)
286	Tolclofos-methyl	14.499	265.0>250.0(15)	265.0>93.0(30)
287	Triadimefon	15.443	208.0>181.0(10)	208.0>111.0(35)
288-1	Triadimenol_1	16.138	130.0>65.0(25)	128.0>65.0(20)
288-2	Triadimenol_2	16.267	130.0>65.0(25)	128.0>65.0(20)
289	Tri-allate	13.742	268.0>226.0(20)	268.0>184.0(10)
290	Triazophos	17.763	161.0>134.0(5)	161.0>106.0(15)
291	Tridiphane	14.718	187.0>159.0(15)	173.0>109.0(30)
292	Trifloxystrobin	17.927	222.0>130.0(10)	116.0>89.0(15)
293	Triflumizole	16.180	278.0>43.0(15)	206>179.0(15)
294	Trifluralin	11.570	306.0>264.0(10)	264.0>160.0(15)
295	Vinclozolin	14.423	198.0>145.0(15)	187.0>124.0(20)
296	Zoxamide	18.530	187.0>159.0(20)	187.0>123.0(20)
