# Supplementary material for: Simultaneous analytical method for 296 pesticide multiresidues in root and rhizome based herbal medicines with GC-MS/MS
Source: PLoS One. 2023 Jul 6;18(7):e0288198. doi: 10.1371/journal.pone.0288198 (PMC10325055; doi:10.1371/journal.pone.0288198)
Supplement: S2 Table — (PDF) [file pone.0288198.s002.pdf]

**S2 Table. The linearities of calibration curves expressed as $r^2$ for the target pesticides in herbal medicines.**

| No. | Pesticide name | Correlation coefficient ($r^2$). | | |
|---|---|---|---|---|
| | | *C. officinale* | *R. glutinosa* | *P. lactiflora* |
| 1 | 2,6-Diisopropylnaphthalene | 0.9885 | 0.9988 | 0.9993 |
| 2 | Acetochlor | 0.9910 | 0.9877 | 0.9857 |
| 3 | EMA | 0.9928 | 0.9721 | 0.9878 |
| 4 | HEMA | 0.9789 | 0.9912 | 0.9817 |
| 5 | Acrinathrin | 0.9971 | 0.9138 | 0.9752 |
| 6 | Alachlor | 0.9997 | 0.9951 | 0.9989 |
| 7 | Aldrin | 0.9987 | 0.9972 | 0.9993 |
| 8 | Dieldrin | 0.9990 | 0.9990 | 0.9880 |
| 9 | Allidochlor | 0.9708 | 0.9997 | 0.9998 |
| 10 | Ametryn | 0.9937 | 0.9997 | 0.9979 |
| 11 | Anilofos | 0.9843 | 0.9991 | 0.9720 |
| 12 | Aramite | 0.9801 | 0.9915 | 0.9919 |
| 13 | Aspon | 0.9771 | 0.9929 | 0.9961 |
| 14 | Atrazine | 0.9705 | 0.9994 | 0.9999 |
| 15 | Azaconazole | 0.9910 | 0.9999 | 0.9990 |
| 16 | Benfluralin | 0.9954 | 0.9949 | 0.9972 |
| 17 | Benfuresate | 0.9878 | 0.9934 | 0.9913 |
| 18 | Benodanil | 0.9810 | 0.9963 | 0.9969 |
| 19 | Benoxacor | 0.9980 | 0.9966 | 0.9991 |
| 20 | Benzoylprop-ethyl | 0.9815 | 0.9755 | 0.9894 |
| 21 | BHC-alpha | 0.9957 | 0.9998 | 0.9994 |
| 22 | BHC-beta | 0.9978 | 0.9921 | 0.9855 |
| 23 | BHC-delta | 0.9982 | 0.9969 | 0.9929 |
| 24 | BHC-gamma | 0.9998 | 0.9981 | 0.9932 |
| 25 | Bifenox | 0.9991 | 0.9989 | 0.9987 |
| 26 | Bifenthrin | 0.9972 | 0.9991 | 0.9981 |
| 27 | Binapacryl | 0.9957 | 0.9992 | 0.9982 |
| 28 | Boscalid | 0.9998 | 0.9982 | 0.9990 |
| 29 | Bromobutide | 0.9878 | 0.9913 | 0.9876 |
| 30 | Bromophos-ethyl | 0.9949 | 1.0000 | 0.9996 |
| 31 | Bromophos-methyl | 0.9917 | 0.9820 | 0.9902 |
| 32 | Bromopropylate | 0.9908 | 0.9977 | 0.9991 |
| 33 | Bupirimate | 0.9997 | 0.9999 | 0.9094 |
| 34 | Buprofezin | 0.9997 | 0.9993 | 0.9994 |
| 35 | Butachlor | 0.9987 | 0.9940 | 0.9944 |
| 36 | Butafenacil | 0.9934 | 0.9667 | 0.9931 |
| 37 | Butralin | 0.9995 | 0.9986 | 0.9977 |
| 38 | Butylate | 0.9967 | 0.9975 | 0.9995 |
| 39 | Cadusafos | 0.9979 | 0.9983 | 0.9993 |
| 40 | Carbophenothion | 0.9370 | 0.9965 | 0.9998 |

| No. | Pesticide name | Correlation coefficient ($r^2$). | | |
|---|---|---|---|---|
| | | *C. officinale* | *R. glutinosa* | *P. lactiflora* |
| 41 | Carboxin | 0.9763 | 0.9991 | 0.9123 |
| 42 | Carfentrazone-ethyl | 0.9989 | 0.9993 | 0.9910 |
| 43 | Chinomethionat | 0.9994 | 0.9996 | 0.9984 |
| 44 | Chlorbenside | 0.9971 | 0.9949 | 0.9908 |
| 45 | Chlorbufam | 0.9757 | 0.9995 | 0.9995 |
| 46 | Chlordane | 0.9997 | 0.9980 | 0.9881 |
| 47 | Chlorethoxyfos | 0.9992 | 0.9932 | 0.9784 |
| 48 | Chlorfenapyr | 0.9849 | 0.9967 | 0.9989 |
| 49 | Chlorfenson | 0.9971 | 0.9932 | 0.9885 |
| 50 | Chlorflurenol-methyl | 0.9998 | 0.9991 | 0.9992 |
| 51 | Chlornitrofen | 0.9997 | 0.9993 | 0.9997 |
| 52 | Chlorobenzilate | 0.9928 | 0.9891 | 0.9973 |
| 53 | Chloroneb | 0.9956 | 0.9938 | 0.9706 |
| 54 | Chloropropylate | 0.9997 | 0.9993 | 0.9994 |
| 55 | Chlorothalonil | 0.9987 | 0.9940 | 0.9944 |
| 56 | Chlorpropham | 0.9934 | 0.9667 | 0.9931 |
| 57 | Chlorpyrifos | 0.9995 | 0.9986 | 0.9977 |
| 58 | Chlorpyrifos-methyl | 0.9967 | 0.9975 | 0.9995 |
| 59 | Chlorthal -dimethyl | 0.9979 | 0.9983 | 0.9993 |
| 60 | Chlorthion | 0.9943 | 0.9917 | 0.9937 |
| 61 | Chlorthiophos | 1.0000 | 1.0000 | 0.9957 |
| 62 | Chlozolinate | 0.9990 | 0.9989 | 0.9992 |
| 63 | Cinidon-ethyl | 0.9704 | 0.9853 | 0.9886 |
| 64 | Cinmethylin | 0.9953 | 0.9995 | 0.9913 |
| 65 | Clomazone | 0.9929 | 0.9717 | 0.9928 |
| 66 | Coumaphos | 0.9415 | 0.9975 | 0.9960 |
| 67 | Cyanophos | 0.9370 | 0.9965 | 0.9998 |
| 68 | Cyflufenamid | 0.9763 | 0.9991 | 0.9123 |
| 69 | Cyfluthrin | 0.9989 | 0.9993 | 0.9910 |
| 70 | Cyhalofop-buthyl | 0.9964 | 0.9973 | 0.9989 |
| 71 | Cyhalothrin | 0.9855 | 0.9996 | 0.9943 |
| 72 | Cypermethrin | 0.9986 | 0.9964 | 0.9854 |
| 73 | Cyprazine | 0.9989 | 0.9999 | 0.9976 |
| 74 | Cyprodinil | 0.9860 | 0.9888 | 0.9984 |
| 75 | DDD (p,p) | 0.9843 | 0.9914 | 0.9649 |
| 76 | DDE (p,p) | 0.9945 | 0.9997 | 0.9972 |
| 77 | DDT (o,p) | 0.9849 | 0.9967 | 0.9989 |
| 78 | DDT (p,p) | 0.9971 | 0.9932 | 0.9885 |
| 79 | Deltamethrin | 0.9998 | 0.9991 | 0.9992 |
| 80 | Tralomethrin | 0.9997 | 0.9993 | 0.9997 |
| 81 | Desmetryn | 0.9928 | 0.9891 | 0.9973 |
| 82 | Dialifos | 0.9968 | 0.9899 | 0.9900 |
| 83 | Di-allate | 0.9984 | 0.9999 | 0.9953 |

| No. | Pesticide name | Correlation coefficient ($r^2$). | | |
|---|---|---|---|---|
| | | *C. officinale* | *R. glutinosa* | *P. lactiflora* |
| 84 | Diazinon | 0.9615 | 0.9621 | 0.9808 |
| 85 | Dichlobenil | 0.9979 | 0.9982 | 0.9986 |
| 86 | Dichlofenthion | 0.9948 | 0.9974 | 0.9997 |
| 87 | Dichlofluanid | 0.9715 | 0.9879 | 0.9972 |
| 88 | Dichlormid | 0.9999 | 0.9998 | 0.9978 |
| 89 | Diclobutrazol | 0.9868 | 0.9939 | 0.9998 |
| 90 | Diclofop-methyl | 0.9910 | 0.9999 | 0.9990 |
| 91 | Dicloran | 0.9954 | 0.9949 | 0.9972 |
| 92 | Dicofol | 0.9878 | 0.9934 | 0.9913 |
| 93 | Dicrotophos | 0.9810 | 0.9963 | 0.9969 |
| 94 | Diethatyl-ethyl | 0.9980 | 0.9966 | 0.9991 |
| 95 | Diethofencarb | 0.9815 | 0.9755 | 0.9894 |
| 96 | Difenoconazole | 0.9957 | 0.9998 | 0.9994 |
| 97 | Diflufenican | 0.9990 | 0.9996 | 0.9997 |
| 98 | Dimepiperate | 0.9300 | 0.9987 | 0.9998 |
| 99 | Dimethachlor | 0.9935 | 0.9988 | 0.9188 |
| 100 | Dimethametryn | 0.9901 | 0.9992 | 0.9972 |
| 101 | Dimethenamid | 0.9949 | 0.9972 | 0.9959 |
| 102 | Dimethipin | 0.9964 | 0.9973 | 0.9989 |
| 103 | Dimethomorph (E,Z) | 0.9855 | 0.9996 | 0.9943 |
| 104 | Dimethylvinphos (E.Z) | 0.9986 | 0.9964 | 0.9854 |
| 105 | Diniconazole | 0.9860 | 0.9888 | 0.9984 |
| 106 | Dinitramine | 0.9843 | 0.9914 | 0.9649 |
| 107 | Dioxathion | 0.9945 | 0.9997 | 0.9972 |
| 108 | Diphenamid | 0.9758 | 0.9994 | 0.9952 |
| 109 | Diphenylamine | 0.9978 | 0.9998 | 0.9901 |
| 110 | Dithiopyr | 0.9914 | 0.9987 | 0.9980 |
| 111 | Edifenphos | 0.9971 | 0.9949 | 0.9908 |
| 112 | Endosulfan-alpha | 0.9870 | 0.9995 | 0.9995 |
| 113 | Endosulfan-beta | 0.9849 | 0.9967 | 0.9738 |
| 114 | Endosulfan-sulfate | 1.0000 | 0.9999 | 0.9932 |
| 115 | Endrin | 0.9955 | 0.9755 | 0.9725 |
| 116 | Endrin-ketone | 0.9803 | 0.9989 | 0.9933 |
| 117 | EPN | 0.9995 | 0.9985 | 0.9996 |
| 118 | Epoxiconazole | 0.9908 | 0.9974 | 0.9104 |
| 119 | EPTC | 0.9996 | 0.9994 | 0.9999 |
| 120 | Etaconazole | 0.9943 | 0.9917 | 0.9937 |
| 121 | Ethalfluralin | 0.9990 | 0.9989 | 0.9992 |
| 122 | Ethion | 0.9704 | 0.9853 | 0.9886 |
| 123 | Ethofumesate | 0.9953 | 0.9995 | 0.9913 |
| 124 | Ethoprophos | 0.9929 | 0.9717 | 0.9928 |
| 125 | Ethychlozate | 0.9415 | 0.9975 | 0.9960 |
| 126 | Etoxazole | 0.9370 | 0.9965 | 0.9998 |

| No. | Pesticide name | Correlation coefficient ($r^2$). | | |
|---|---|---|---|---|
| | | *C. officinale* | *R. glutinosa* | *P. lactiflora* |
| 127 | Etridiazole | 0.9763 | 0.9991 | 0.9123 |
| 128 | Fenamidone | 0.9989 | 0.9993 | 0.9910 |
| 129 | Fenarimol | 0.9994 | 0.9996 | 0.9984 |
| 130 | Fenbuconazole | 0.9997 | 0.9980 | 0.9881 |
| 131 | Fenchlorphos | 0.9992 | 0.9850 | 0.9868 |
| 132 | Fenclorim | 0.9992 | 0.9932 | 0.9784 |
| 133 | Fenfuram | 0.9849 | 0.9967 | 0.9989 |
| 134 | Fenitrothion | 0.9971 | 0.9932 | 0.9885 |
| 135 | Fenobucarb | 0.9998 | 0.9991 | 0.9992 |
| 136 | Fenothiocarb | 0.9997 | 0.9993 | 0.9997 |
| 137 | Fenoxanil | 0.9928 | 0.9891 | 0.9973 |
| 138 | Fenpropathrin | 0.9968 | 0.9899 | 0.9900 |
| 139 | Fenpropimorph | 0.9984 | 0.9999 | 0.9953 |
| 140 | Fenpyrazamine | 0.9615 | 0.9621 | 0.9808 |
| 141 | Fenson | 0.9979 | 0.9982 | 0.9986 |
| 142 | Fenthion | 0.9948 | 0.9974 | 0.9997 |
| 143 | Fenvalerate | 0.9967 | 0.9988 | 0.9980 |
| 144 | Fipronil | 0.9996 | 0.9986 | 0.9991 |
| 145 | Flamprop-isopropyl | 0.9783 | 0.9762 | 0.9835 |
| 146 | Fluacrypyrim | 0.9983 | 0.9970 | 0.9958 |
| 147 | Fluazifop-butyl | 0.9912 | 0.9977 | 0.9987 |
| 148 | Fluchloralin | 0.9995 | 0.9218 | 0.9138 |
| 149 | Flucythrinate | 0.9713 | 0.9725 | 0.9813 |
| 150 | Fluensulfone | 0.9949 | 0.9939 | 0.9898 |
| 151 | Flufenpyr-ethyl | 0.9972 | 0.9879 | 0.9715 |
| 152 | Flumetralin | 0.9978 | 0.9998 | 0.9999 |
| 153 | Flumioxazine | 0.9993 | 0.9988 | 0.9885 |
| 154 | Fluopyram | 0.9857 | 0.9877 | 0.9910 |
| 155 | Flurochloridone | 0.9878 | 0.9721 | 0.9928 |
| 156 | Fluorodifen | 0.9817 | 0.9912 | 0.9789 |
| 157 | Fluquinconazole | 0.9752 | 0.9138 | 0.9971 |
| 158 | Flurtamone | 0.9989 | 0.9951 | 0.9997 |
| 159 | Flusilazole | 0.9993 | 0.9972 | 0.9987 |
| 160 | Flutianil | 0.9880 | 0.9990 | 0.9990 |
| 161 | Fluvalinate | 0.9986 | 0.9977 | 0.9973 |
| 162 | Fluxapyroxad | 0.9998 | 0.9999 | 0.9992 |
| 163 | Fonofos | 0.9996 | 0.9911 | 0.9990 |
| 164 | Formothion | 0.9706 | 0.9938 | 0.9956 |
| 165 | Fthalide | 0.9994 | 0.9993 | 0.9997 |
| 166 | Halfenprox | 0.9944 | 0.9940 | 0.9987 |
| 167 | Heptachlor | 0.9931 | 0.9667 | 0.9934 |
| 168 | Heptachlor epoxide | 0.9977 | 0.9986 | 0.9995 |
| 169 | Heptenophos | 0.9995 | 0.9975 | 0.9967 |

| No. | Pesticide name | Correlation coefficient ($r^2$). | | |
|---|---|---|---|---|
| | | *C. officinale* | *R. glutinosa* | *P. lactiflora* |
| 170 | Hexachlorbenzene | 0.9993 | 0.9983 | 0.9979 |
| 171 | Hexythiazox | 0.9982 | 0.9992 | 0.9957 |
| 172 | Indanofan | 0.9990 | 0.9982 | 0.9998 |
| 173 | Indoxacarb | 0.9876 | 0.9913 | 0.9878 |
| 174 | Ipconazole | 0.9996 | 1.0000 | 0.9949 |
| 175 | Iprobenfos | 0.9902 | 0.9820 | 0.9917 |
| 176 | Iprodione | 0.9991 | 0.9977 | 0.9908 |
| 177 | Isazofos | 0.9094 | 0.9999 | 0.9997 |
| 178 | Isofenphos | 0.9962 | 0.9998 | 0.9998 |
| 179 | Isofenphos-methyl | 0.9995 | 0.9993 | 0.9998 |
| 180 | Isoprocarb | 0.9871 | 1.0000 | 0.9991 |
| 181 | Isopropalin | 0.9783 | 0.9961 | 0.9971 |
| 182 | Isoprothiolane | 0.9983 | 0.9991 | 0.9995 |
| 183 | Isopyrazam | 0.9911 | 0.9756 | 0.9801 |
| 184 | Isotianil | 0.9867 | 0.9893 | 0.9729 |
| 185 | Isoxadifen-ethyl | 0.9978 | 0.9985 | 0.9982 |
| 186 | Kresoxim-methyl | 0.9939 | 0.9884 | 0.9964 |
| 187 | Leptophos | 0.9990 | 0.9988 | 0.9992 |
| 188 | Mefenpyr-diethyl | 0.9909 | 0.9878 | 0.9857 |
| 189 | Mepanipyrim | 0.9998 | 0.9997 | 0.9708 |
| 190 | Mepronil | 0.9979 | 0.9997 | 0.9937 |
| 191 | Metalaxyl | 0.9843 | 0.9720 | 0.9991 |
| 192 | Methidathion | 0.9801 | 0.9919 | 0.9915 |
| 193 | Methoprotryne | 0.9771 | 0.9961 | 0.9929 |
| 194 | Methoxychlor | 0.9705 | 0.9999 | 0.9994 |
| 195 | Methyl trithion | 0.9960 | 0.9990 | 0.9959 |
| 196 | Metolachlor | 0.9833 | 0.9959 | 0.9937 |
| 197 | Metribuzin | 0.9982 | 0.9929 | 0.9969 |
| 198 | MGK_264 | 0.9998 | 0.9932 | 0.9981 |
| 199 | Mirex | 0.9991 | 0.9987 | 0.9989 |
| 200 | Molinate | 0.9972 | 0.9981 | 0.9991 |
| 201 | Monolinuron | 1.0000 | 0.9812 | 0.9991 |
| 202 | Myclobutanil | 0.9911 | 0.9995 | 0.9998 |
| 203 | Nitrapyrin | 0.9999 | 0.9994 | 0.9997 |
| 204 | Nitrothal-isopropyl | 0.9993 | 0.9911 | 0.9989 |
| 205 | Nonachlor | 0.9999 | 0.9992 | 0.9999 |
| 206 | Nuarimol | 0.9958 | 0.9913 | 0.9977 |
| 207 | O-Phenylphenol | 0.9997 | 0.9999 | 0.9999 |
| 208 | Oxadiazon | 0.9997 | 0.9983 | 0.9998 |
| 209 | Oxadixyl | 0.9998 | 1.0000 | 1.0000 |
| 210 | Oxyfluorfen | 0.9918 | 0.9983 | 0.9996 |
| 211 | Paclobutrazol | 0.9999 | 0.9979 | 0.9999 |
| 212 | Parathion | 0.9999 | 0.9997 | 0.9998 |

| No. | Pesticide name | Correlation coefficient ($r^2$). | | |
|---|---|---|---|---|
| | | *C. officinale* | *R. glutinosa* | *P. lactiflora* |
| 213 | Parathion-ethyl | 0.9995 | 0.9884 | 0.9940 |
| 214 | Parathion-methyl | 0.9999 | 0.9997 | 0.9998 |
| 215 | Penconazole | 0.9977 | 0.9973 | 0.9993 |
| 216 | Pendimethalin | 0.9961 | 0.9908 | 0.9994 |
| 217 | Penflufen | 0.9975 | 0.9859 | 0.9989 |
| 218 | Pentachlorobenzonitrile | 0.9985 | 0.9979 | 0.9954 |
| 219 | Penthiopyrad | 0.9995 | 0.9998 | 0.9998 |
| 220 | Pentoxazone | 0.9996 | 0.9988 | 0.9997 |
| 221 | Permethrin | 0.9992 | 0.9996 | 1.0000 |
| 222 | Perthane | 0.9997 | 0.9998 | 0.9990 |
| 223 | Phenthoate | 1.0000 | 0.9997 | 0.9997 |
| 224 | Phosalone | 0.9994 | 0.9984 | 0.9990 |
| 225 | Phosmet | 0.9975 | 0.9998 | 0.9999 |
| 226 | Phosphamidon | 0.9983 | 0.9874 | 0.9975 |
| 227 | Picoxystrobin | 0.9829 | 0.9765 | 0.9814 |
| 228 | Piperonyl butoxide | 0.9424 | 0.9987 | 0.9986 |
| 229 | Pirimicarb | 0.9874 | 0.9827 | 0.9704 |
| 230 | Pirimiphos-ethyl | 0.9980 | 0.9988 | 0.9983 |
| 231 | Pirimiphos-methyl | 0.9560 | 0.9499 | 0.9836 |
| 232 | Pretilachlor | 0.9996 | 0.9963 | 0.9992 |
| 233 | Prochloraz | 0.9894 | 0.9786 | 0.9929 |
| 234 | 2,4,6-Trichlorophenol | 0.9756 | 0.9933 | 0.9954 |
| 235 | Procymidone | 0.9928 | 0.9986 | 0.9878 |
| 236 | Prodiamine | 0.9998 | 0.9982 | 0.9997 |
| 237 | Profenofos | 0.9996 | 0.9999 | 0.9999 |
| 238 | Profluralin | 0.9987 | 0.9993 | 0.9999 |
| 239 | Prohydrojasmon | 0.9972 | 1.0000 | 0.9981 |
| 240 | Prometon | 0.9993 | 0.9905 | 1.0000 |
| 241 | Prometryn | 0.9880 | 0.9998 | 0.9954 |
| 242 | Propachlor | 0.9999 | 0.9999 | 0.9998 |
| 243 | Propanil | 0.9995 | 0.9984 | 0.9999 |
| 244 | Propazine | 0.9938 | 0.9999 | 0.9927 |
| 245 | Propetamphos | 0.9990 | 0.9996 | 0.9997 |
| 246 | Propham | 0.9999 | 0.9993 | 0.9995 |
| 247 | Propiconazole | 0.9996 | 0.9873 | 0.9994 |
| 248 | Propisochlor | 0.9978 | 0.9981 | 0.9895 |
| 249 | Propyzamide | 0.9991 | 0.9974 | 0.9999 |
| 250 | Prothiofos | 0.9969 | 0.9984 | 0.9962 |
| 251 | Pyracarbolid | 0.9953 | 0.9994 | 0.9996 |
| 252 | Pyraclofos | 0.9978 | 0.9998 | 0.9995 |
| 253 | Pyraflufen-ethyl | 0.9994 | 0.9995 | 0.9998 |
| 254 | Pyrazophos | 0.9986 | 0.9998 | 0.9998 |
| 255 | Pyridalyl | 0.9994 | 0.9986 | 0.9998 |

| No. | Pesticide name | Correlation coefficient ($r^2$). | | |
|---|---|---|---|---|
| | | *C. officinale* | *R. glutinosa* | *P. lactiflora* |
| 256 | Pyrifenox | 0.9985 | 0.9986 | 0.9992 |
| 257 | Pyriftalid | 0.9997 | 0.9961 | 0.9995 |
| 258 | Pyrimethanil | 0.9908 | 0.9975 | 0.9994 |
| 259 | Pyriminobac-methyl | 0.9959 | 0.9985 | 0.9989 |
| 260 | Quinalphos | 0.9998 | 0.9996 | 0.9998 |
| 261 | Quinoxyfen | 0.9988 | 0.9992 | 0.9997 |
| 262 | Quintozene | 0.9996 | 0.9997 | 1.0000 |
| 263 | Quizalofop-ethyl | 0.9979 | 0.9997 | 0.9990 |
| 264 | Silafluofen | 0.9998 | 1.0000 | 0.9990 |
| 265 | Simeconazole | 0.9997 | 0.9994 | 0.9997 |
| 266 | Simetryn | 0.9984 | 0.9943 | 0.9990 |
| 267 | Spiromesifen | 0.9937 | 1.0000 | 0.9917 |
| 268 | Spiroxamine | 0.9957 | 0.9990 | 1.0000 |
| 269 | Sulfotep | 0.9886 | 0.9953 | 0.9853 |
| 270 | Tebuconazole | 0.9913 | 0.9929 | 0.9995 |
| 271 | Tebufenpyrad | 0.9928 | 0.9415 | 0.9717 |
| 272 | Tebupirimfos | 0.9960 | 0.9945 | 0.9975 |
| 273 | Tecnazene | 0.9822 | 0.9996 | 0.9965 |
| 274 | Tefluthrin | 0.9763 | 0.9894 | 0.9991 |
| 275 | Terbacil | 0.9989 | 0.9756 | 0.9993 |
| 276 | Terbumeton | 0.9964 | 0.9928 | 0.9973 |
| 277 | Terbutryn | 0.9855 | 0.9998 | 0.9996 |
| 278 | Tetrachlorvinphos | 0.9167 | 0.9996 | 0.9228 |
| 279 | Tetraconazole | 0.9986 | 0.9987 | 0.9964 |
| 280 | Tetradifon | 0.9989 | 0.9972 | 0.9999 |
| 281 | Tetramethrin | 0.9860 | 0.9993 | 0.9888 |
| 282 | Tetrasul | 0.9945 | 0.9999 | 0.9997 |
| 283 | Thifluzamide | 0.9849 | 0.9995 | 0.9967 |
| 284 | Thiometon | 0.9997 | 0.9938 | 0.9993 |
| 285 | Thionazin | 0.9987 | 0.9990 | 0.9940 |
| 286 | Tolclofos-methyl | 0.9934 | 0.9999 | 0.9667 |
| 287 | Triadimefon | 0.9995 | 0.9996 | 0.9986 |
| 288 | Triadimenol | 0.9967 | 0.9754 | 0.9975 |
| 289 | Tri-allate | 0.9929 | 0.9991 | 0.9717 |
| 290 | Triazophos | 0.9415 | 0.9969 | 0.9975 |
| 291 | Tridiphane | 0.9370 | 0.9953 | 0.9965 |
| 292 | Trifloxystrobin | 0.9763 | 0.9978 | 0.9991 |
| 293 | Triflumizole | 0.9989 | 0.9994 | 0.9993 |
| 294 | Trifluralin | 0.9994 | 0.9986 | 0.9996 |
| 295 | Vinclozolin | 0.9971 | 0.9994 | 0.9949 |
| 296 | Zoxamide | 0.9957 | 0.9985 | 0.9995 |
