# Supplementary material for: Simultaneous analytical method for 296 pesticide multiresidues in root and rhizome based herbal medicines with GC-MS/MS
Source: PLoS One. 2023 Jul 6;18(7):e0288198. doi: 10.1371/journal.pone.0288198 (PMC10325055; doi:10.1371/journal.pone.0288198)
Supplement: S3 Table — (PDF) [file pone.0288198.s003.pdf]

S3 Table. Quantitation results of pesticide multiresidues in *C. officinale*, *R. glutinosa*, and *P. lactiflora* obtained from commercial markets.

***C. officinale* from Republic of Korea (A1 to A14)**														(μg/kg)
Name	A1	A2	A3	A4	A5	A6	A7	A8	A9	A10	A11	A12	A13	A14
Bifenthrin	-	-	21.9	-	-	-	10.5	-	24.1	-	31.5	-	25.5	24.4
Chlorpyrifos	-	-	-	21.5	-	15.2	24.1	25.9	-	29.2	-	-	41.2	-
Difenoconazole	-	-	21.5	52.2	33.1	-	28.1	31.2	-	-	32.1	-	35.1	-
Dimethomorph	-	-	89.2	140.5	-	90.1	-	150.5	50.2	-	139.2	-	-	-
Diniconazole	-	-	-	30.9	-	-	-	33.3	-	-	-	-	32,9	-
Metribuzin	-	-	-	-	19.5	-	18.5	-	-	-	-	21.8	-	-
Pendimethalin	-	-	-	-	86.5	-	110.5	90.5	-	-	70.5	-	52.1	-
Quinalphos	-	-	-	10.5	-	-	11.9	15.8	-	21.5	-	24.5	-	-
Tebuconazole	-	-	28.9	-	-	52.5	-	32.5	-	-	21.5	-	-	-
Tebufenpyrad	-	-	-	9.7	-	-	32.5	-	-	21.9	-	-	-	-
***C. officinale* from China (B1 to B5)**														(μg/kg)
Not detected in all samples														

***R. glutinosa* from Republic of Korea (C1 to C8)**								(μg/kg)
Name	C1	C2	C3	C4	C5	C6	C7	C8
Dimethomorph	-	285.1	58.2	-	100.5	85.2	321.5	-
***R. glutinosa* from China (D1 to D7)**								(μg/kg)
Not detected in all samples								
***P. lactiflora* from Republic of Korea (E1 to E10)**								(μg/kg)
Not detected in all samples								
***P. lactiflora* from China (F1 to F3)**								(μg/kg)
Not detected in all samples								
