# Supplementary material for: Simultaneous analytical method for 296 pesticide multiresidues in root and rhizome based herbal medicines with GC-MS/MS
Source: PLoS One. 2023 Jul 6;18(7):e0288198. doi: 10.1371/journal.pone.0288198 (PMC10325055; doi:10.1371/journal.pone.0288198)
Supplement: S1 Fig — The average relative intensity in unpulsed injection was set to 100%. (PDF) [file pone.0288198.s004.pdf]

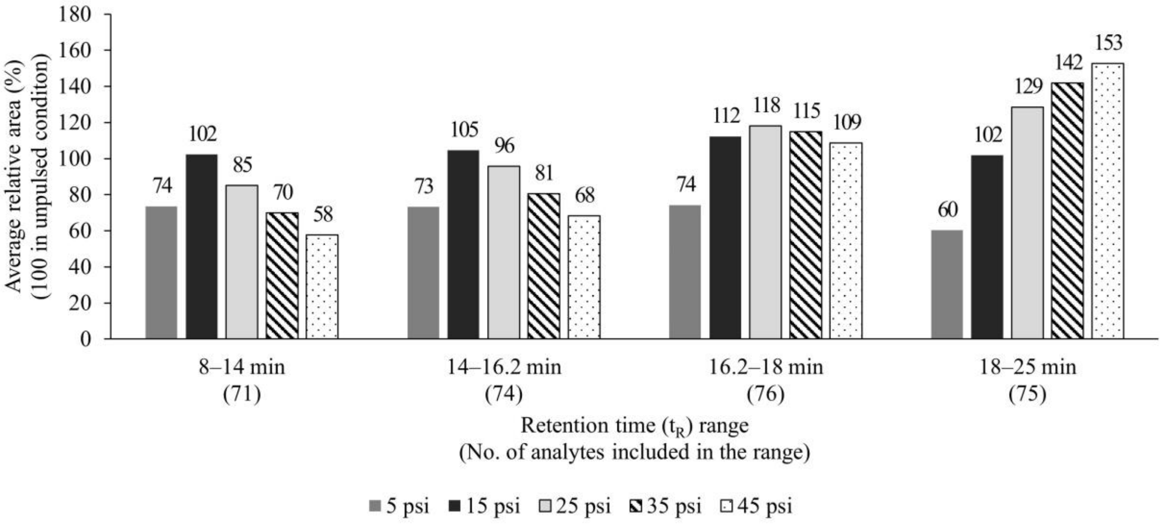

S1 Fig. The average relative intensity (area) of target pesticides grouped by the four retention time (t_R) segments (8–14, 14–16.2, 16.2–18, and 18–25 min). The average relative intensity in unpulsed injection was set to 100%.
