# Supplementary material for: Simultaneous analytical method for 296 pesticide multiresidues in root and rhizome based herbal medicines with GC-MS/MS
Source: PLoS One. 2023 Jul 6;18(7):e0288198. doi: 10.1371/journal.pone.0288198 (PMC10325055; doi:10.1371/journal.pone.0288198)
Supplement: S2 Fig — In cases where pesticides were not detected in certain methods and no RSD data was available, they were excluded from the statistics. (PDF) [file pone.0288198.s005.pdf]

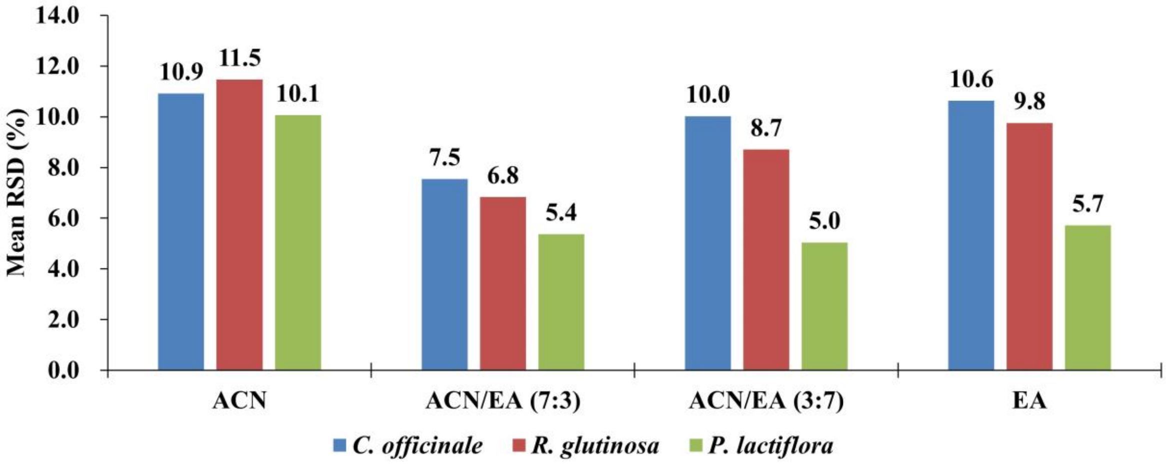

S2 Fig. The means of relative standard deviations (RSDs) for recoveries of 296 pesticides in *C. officinale*, *R. glutinosa*, and *P. lactiflora* under the extraction conditions of ACN, ACN/EA (7:3, v/v), ACN/EA (3:7), and EA. In cases where pesticides were not detected in certain methods and no RSD data was available, they were excluded from the statistics.
