# Supplementary material for: Simultaneous analytical method for 296 pesticide multiresidues in root and rhizome based herbal medicines with GC-MS/MS
Source: PLoS One. 2023 Jul 6;18(7):e0288198. doi: 10.1371/journal.pone.0288198 (PMC10325055; doi:10.1371/journal.pone.0288198)
Supplement: S3 Fig — (PDF) [file pone.0288198.s006.pdf]

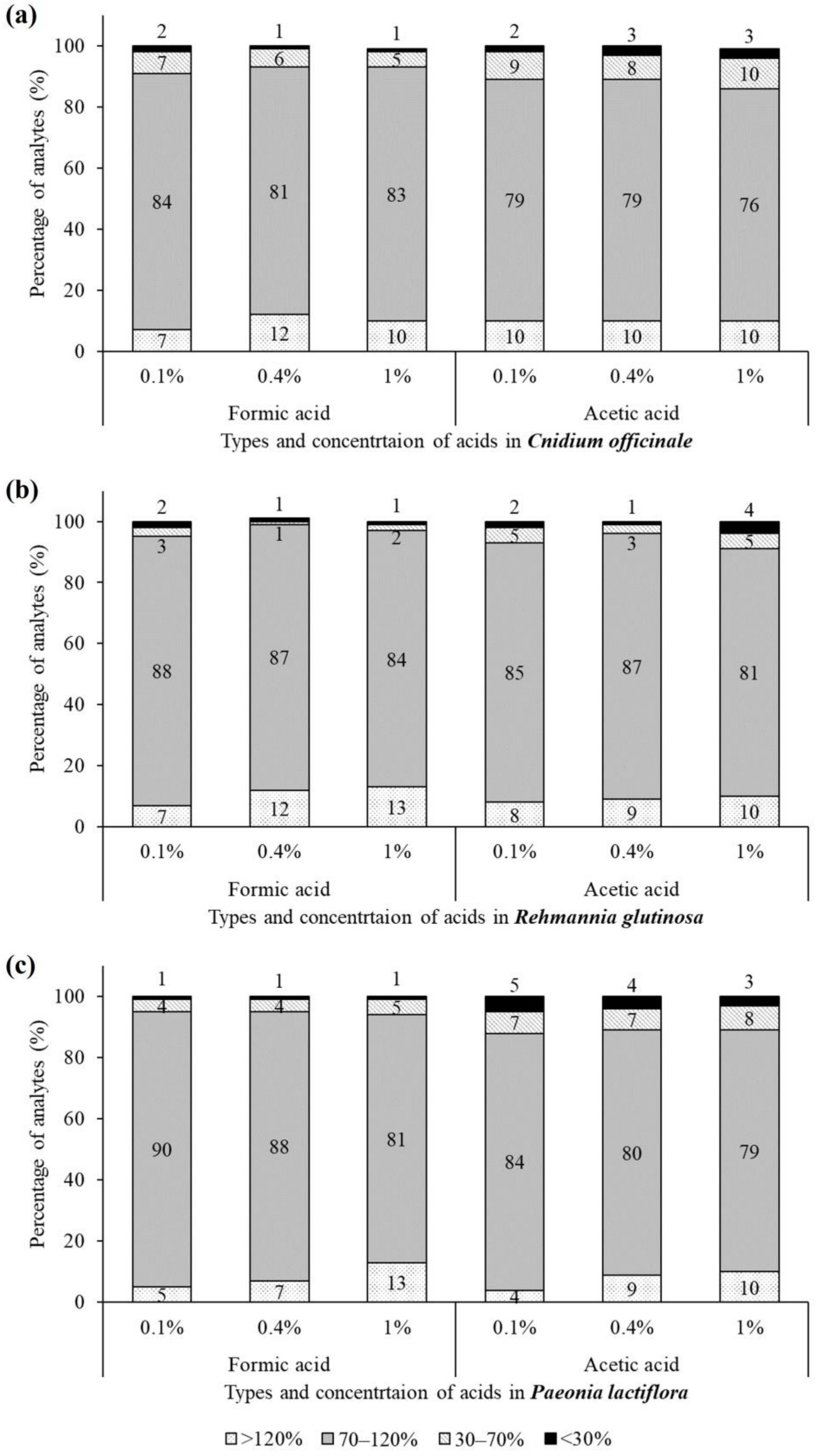

S3 Fig. Distributions of recovery ranges of target pesticides when using 0.1, 0.4, and 1% formic acid or acetic acid in ACN/EA (7:3, v/v).
