# Supplementary material for: Simultaneous analytical method for 296 pesticide multiresidues in root and rhizome based herbal medicines with GC-MS/MS
Source: PLoS One. 2023 Jul 6;18(7):e0288198. doi: 10.1371/journal.pone.0288198 (PMC10325055; doi:10.1371/journal.pone.0288198)
Supplement: S4 Fig — Control (pesticide-free) samples of (a) C. officinale, (b) R. glutinosa, and (c) P. lactiflora were analyzed after preparation using the established method. (PDF) [file pone.0288198.s007.pdf]

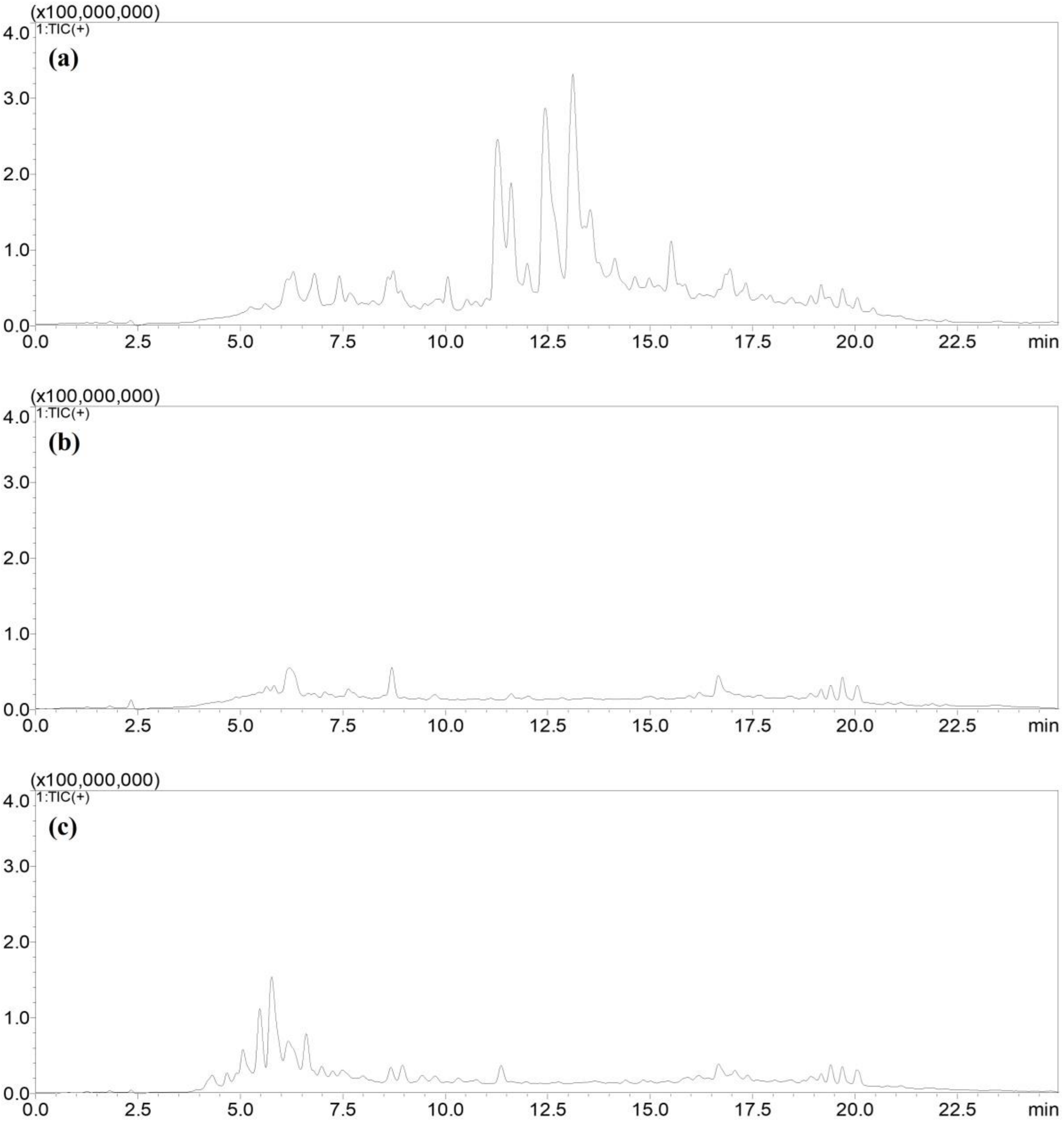

S4 Fig. Total ion chromatograms (TICs) through full scan analysis (m/z range 50–500). Control (pesticide-free) samples of (a) *C. officinale*, (b) *R. glutinosa*, and (c) *P. lactiflora* were analyzed after preparation using the established method.
