# Supplementary material for: Simultaneous analytical method for 296 pesticide multiresidues in root and rhizome based herbal medicines with GC-MS/MS
Source: PLoS One. 2023 Jul 6;18(7):e0288198. doi: 10.1371/journal.pone.0288198 (PMC10325055; doi:10.1371/journal.pone.0288198)
Supplement: Attachment — Submitted filename: PLOS ONE_Yang_Response to reviewer comments.docx [file pone.0288198.s009.docx]

**<Author’s Response to Reviewer Comments for “PONE-D-22-33518”>**

**Academic Editor:**

1. methodologically: it is essential to better describe the "pesticide-free" reference matrix. How was it qualified as pesticide-free? What method was used to check this? How is it produced? These elements of precision are key technical points for the validation of the detection method.

**Answer**: Thank you for your valuable comments. We were unable to confirm from the manufacturer how it was produced, but we confirmed the absence of pesticides in the samples using three established versions of the QuEChERS analysis method. We included this information in the Materials and Methods section (Lines 117-118 in Track Changes version). “Pesticides were confirmed to be absent in these samples using three different versions of the QuEChERS method [13, 21, 23].”

2. at the level of the discussion: this one will have to be deepened to put in perspective the measured levels of contaminants and/or residues with the literature, with the possible available reference values and/or with the possible regulatory values (maximum residual limits, acceptable daily dose). This reflection is essential to determine if the levels observed are significant and could have a health impact.

**Answer**: Thank you for your insightful feedback. For MRL, it is not established for the three herbs in the Republic of Korea nor China. Therefore, we used database of acceptable criteria of the Ministry of Food and Drug Safety of the Republic of Korea, which is based on the ADI and the daily intake of the corresponding herbal medicine. As a result, most of pesticides in the samples showed lower levels than those of acceptable criteria. The residue results can be used as reference data for the pesticide risk assessment in herbal medicines. We described this information in Lines 425-431 of the manuscript.

**Reviewer #1:**

1. Please refer to the attached file for comment. I used the tracking function.

**Answer**: Thank you for your valuable comments. Based on your suggestion, we correct the manuscript and revised parts were highlighted in red color as well as tracked changes.

2. Main Comment: The pesticide detection rate is very high. Is PHI and MRL set for the detected pesticide? The author should consider using this.

**Answer**: Thank you for your valuable comments. In the Ministry of Food and Drug Safety of the Republic of Korea, acceptable criteria of the pesticide residues in herbal medicines have been established based on the acceptable daily intake (ADI) and the daily intake of the corresponding herbal medicine. We showed the acceptable criteria in Table 5 and found that residue levels of most of pesticides were below acceptable criteria. We described this information in Lines 425-431 of the manuscript (Track Changes version).

**Reviewer #2:**

1. The introduction should describe in more details 1) the choice of the herbal medicines, 2) the choice of the pesticides studied, 3) the rules related to the use of such medicines at least in China and Korea. For example, the maximum residue limits are said to be slightly different in herbal medicines than in common crop but not given. Moreover even if no official rule is said to regulate the targeted compounds, an order of magnitude of the acceptable daily intake of pesticides residues according to the local pharmacopeia would be appreciated.

**Answer**: Thank you for your valuable comments. We described the answer to question in the introduction. 1) the choice of the herbal medicines: Herbal medicines are commonly used in many countries, and the most popular ones in the Republic of Korea are *Cnidium officinale*, *Rehmannia glutinosa*, and *Paeonia lactiflora*. We have already fully explained the reasons for choosing three herbal medicines at Lines 45-52 (Track Changes version). 2) the choice of the pesticides studied: We selected the target pesticides because Most of the targeted pesticides are regulated as MRLs in food in both the Republic of Korea and China. (Lines 88-89) 3) the rules related to the use of such medicines at least in China and Korea: We also include the MRLs of China in Lines 58-60. ADI information of target pesticides is available on the URL:
<https://www.mfds.go.kr/brd/m_1060/view.do?seq=14475&srchFr=&srchTo=&srchWord=&srchTp=&itm_seq_1=0&itm_seq_2=0&multi_itm_seq=0&company_cd=&company_nm=&page=1>

We presented the acceptable criteria for pesticides in herbal medicines based on the ADI in Lines 425-431 of the manuscript.

2. When developing the choice of the extraction solvents, more details on the chemical composition of herbal matrix is needed: why is it so different from common crops?

**Answer**: Thank you for your insightful feedback. Roots and rhizomes of herbal medicines contain more abundant complex matrices that contain many biologically active ingredients, secondary metabolites, and phytochemicals than other foods, however, the co-extraction of these phytochemicals with pesticides during sample preparation can interfere with the accurate determination of pesticide residues. We have added this sentence to manuscript with a reference in Lines 65-71.

3. Acetonitrile is considered as an extraction solvent used in the QuEChERS method whereas strictly speaking it does not allow two phases in an aqueous sample.

**Answer**: Acetonitrile is a polar solvent that exhibits miscibility with water. However, the addition of sodium chloride (NaCl) increases the polarity of water, leading to phase separation from acetonitrile. QuEChERS contains NaCl as a component.

4. The mixture of acetonitrile with an extraction solvent chose as ethyl acetate is expected to combine strength of both solvents: a reference or explanation is needed here.

**Answer**: ACN and EA are well-miscible, and by adjusting their ratio, a desired polar solvent mixture can be obtained. As the reviewer’s valuable opinion, we included the sentence with a reference in Lines 81-82.

5. The material and method part is well described but a doubt remains considering the specificity of the method: the “pesticide-free” herbal medicines are used as a blank matrix. How are these herbal medicines produced? How is the absence of all the 296 pesticides determined at the level of sensitivity described later in the paper. It suggests that the company from which these pesticide-free were purchased is able to quantify the targeted pesticides.

**Answer**: Thank you for your valuable comments. We purchased pesticide-free herbal samples from a company, so we do not know the producing process of these samples. However, we confirmed the absence of pesticides in these samples using three existing representative QuEChERS methods. We have described this in Lines 117-118 of the manuscript.

6. The experimental conditions used for the GC-MS/MS analysis should be referenced.

**Answer**: We modified the GC-MS/MS conditions based on the method of Park et al. (2022), and this information has been included with the reference in Lines 134-135 of the manuscript.

7. p10 - The choice of the ACN/EA ratio should be documented: why 3/7 and not 4/6, 5/5? Is it based from previous work?

**Answer**: Thank you for your insightful feedback. We attempted to find a study on the QuEChERS extraction efficiency of ACN and EA mixture, but there were limited resources available. This study is believed to be the first attempt to compare the extraction solvent using the ACN/EA mixture. Therefore, we carried out the experiment with the four solvent conditions of ACN 100%, ACN/EA (7:3), ACN/EA (3:7), and EA 100% by adjusting the ratio of ACN and EA by 30-40% difference. While it is possible to conduct more detailed experiments with different ratios of ACN and EA, such as 10:0, 8:2, 6:4, 4:6, 2:8, and 0:10, this would greatly increase the number of experiments and it is also expected that there is not much difference between adjacent conditions.

8. p11 - How does the study show that EA is more likely to co-extract interferences that ACN?

**Answer**: We extracted control samples using ACN and EA and measured the weight of dry matters after drying the crude extracts. We found that in all three types of herbal samples, the weight of the dry matters from EA extract was greater than that from the ACN extract, confirming that co-extract was highly contained. We have summarized this result and presented it in Fig 1.

9. p11 - Figures are blurred and red could not be seen apart from SI ones.

**Answer**: Thank you for your valuable comments. In Fig 2, we increased the size of the text and used bold font and changed the red dotted line to black dotted line to make it more visible.

10. p11 - Tiometon is described but not visible from fig. 2

**Answer**: As the reviewer’s valuable mention, Thiometon was added in Fig 2 a. We have included "Fig 2 a, Fig 2 b, and Fig 2 c" in the sentences on Lines 249 and 252 to make it clear which parts of the figures the sentences are explaining.

11. figS2 shows a mean relative standard deviation over the 296 pesticides but no error bars are seen

**Answer**: Yes, Fig S2 shows a mean RSD for recoveries of the 296 pesticides. The RSD values of the individual pesticides are varied widely. Even some pesticides were not detected in certain methods, thus excluded from the statistics. Therefore, the error bars were not used in this figure, so they are included in the supporting information to verify the overall trends of repeatability for each preparation. In order to provide a clear explanation of the figure, we have added the sentence "In cases where pesticides were not detected in certain methods and no RSD data was available, they were excluded from the statistics." at the end of the caption.

12. p15 - The method validation should be referenced or described in more details as only the performances in terms of LOQ is here given. If the MRL are available for some compounds, they should be given or referenced.

**Answer**: As the reviewer’s valuable mention, we have included the following sentence along with the reference (SANTE/12682/2019) for the analytical method in Lines 326-329: “The established analytical method for the 296 target pesticides underwent validation using three parameters, including LOQ, linearity of calibration, and recovery. The method was evaluated in accordance with SANTE/12682/2019 guidelines [30].”

13. p22 – is FigS4 meant after the whole treatment?

**Answer**: Yes, FigS4 is typically meant to be viewed after the entire treatment. In order to provide a clear explanation of the figure, we have added the words "after preparation using the established method." at the end of the caption.

14. The method is finally applied to samples obtained from commercial markets with nice performances. It highlights that and the 296 targeted compounds, a maximum of 10 where detected beyond the LOQ in of the herbal medicine. More details would be appreciated here about the origin of the samples: not only the country but also the method of cultivation, of sampling, of storage...are these results of concern in terms of health risk?

**Answer**: Thank you for your valuable opinion. We were unable to obtain information about the cultivation, sampling, and storage of herbal samples as we purchased all the samples from the commercial markets. The aim of this study is to apply the novel analytical method to real samples, and the method will be used as a standard method in residue studies of crops to establish MRLs and PHIs of pesticide in herbal samples.
