# Supplementary material for: Simultaneous analytical method for 296 pesticide multiresidues in root and rhizome based herbal medicines with GC-MS/MS
Source: PLoS One. 2023 Jul 6;18(7):e0288198. doi: 10.1371/journal.pone.0288198 (PMC10325055; doi:10.1371/journal.pone.0288198)
Supplement: Attachment — Submitted filename: PLOS ONE_Yang_Response to reviewer comments.docx [file pone.0288198.s010.docx]

**<Author’s Response to Reviewer Comments for “PONE-D-22-33518R1”>**

**Journal Requirements:**

1. Please review your reference list to ensure that it is complete and correct. If you have cited papers that have been retracted, please include the rationale for doing so in the manuscript text, or remove these references and replace them with relevant current references. Any changes to the reference list should be mentioned in the rebuttal letter that accompanies your revised manuscript. If you need to cite a retracted article, indicate the article’s retracted status in the References list and also include a citation and full reference for the retraction notice.

**Answer**: Thank you for your advice regarding the references. Upon review, all references, except for reference number 6, are correct and up-to-date. For reference number 6, the original URL was expired, so we replaced it with the current, valid URL. We have carefully ensured no retracted papers were cited without proper indication. The detailed changes are noted in the attached rebuttal letter with our revised manuscript.

**Academic Editor:**

1. Thorough English editing is needed. Difficult to follow the write up. For example, see the line number 29-30 in abstract. I am unable to comprehend.

**Answer**: Thank you for your valuable comments. Our draft manuscript was proofread by Editage, a renowned English editing service. However, we recognized that there were still some awkward sentences. Therefore, we have entrusted our manuscript to an English language expert and have undertaken a thorough re-editing process, including line 29-30, to improve the overall clarity of the text.

2. MM section needs to be rewritten:

a. What is n=9, n=3 so on? Elaborate.

b. Clean up agents: GCB in dspe tube as mentioned in materials/reagents does not match in this section.

c. A proper description on HLB cartridge should be given. How were they preconditioned? What was the suction? Which solvent was used to elute etc. Please see any previous paper.

d. In sample preparation, both cartridge and alumina were used? Nothing is mentioned in MM. Should be mentioned here. Why it was chosen? with whom it was compared?

e. Before injection to GCMS, the centrifuged material should have been filtered.

f. Please clarify how are going to get a concentration of 2.5 ng/ml in extract at 10 ppb fortification.

g. Why you have not used any water to saturate the sample? There are several advantages of using water. In addition, the recovery of extract (ACN/EA) will be very less in dry sample.

h. Nothing is mentioned on Matrix of three medicinal plants in MM section. whereas, most of the RD section is comparison of the three matrices.

i. Any statistics

**Answer**: Thank you for your advice regarding the MM section.

a. The "*n* = 9" and "*n* = 3" in the text represent the number of repetitions for each experiment. To clarify this point, I have revised the paragraph in Lines 163-164 (This process was repeated nine times (*n* = 9) for each type of solvent.) and Lines 169-170 (This study was also repeated three times (*n* = 3) for each type of samples.).

b. Thank you for your careful reading and for pointing out the discrepancy related to the amount of GCB in d-SPE tubes mentioned in different sections of the manuscript. Upon reviewing, we found that the correct amounts of GCB are as stated in the first section of the manuscript, which are 2.5 mg and 7.5 mg for tubes 5982-5221 and 5982-5321 respectively. Therefore, the respective sentences in the second section in Lines (180-181) "The extracted samples from the optimized extraction step were purified using various types of d-SPE sorbents... (5) 25 mg PSA and 25 mg GCB, (6) 50 mg PSA and 50 mg GCB..." should be corrected as follows: "(5) 25 mg PSA and 2.5 mg GCB, (6) 25 mg PSA and 7.5 mg GCB."

c. In response to your query about the HLB cartridge, we used the Oasis PRiME HLB cartridge “Plus Light” in our study. This is a filter-type cartridge (R-Figure 1), which differs from the traditional cylindrical-type Oasis PRiME HLB. It specifically designed to simplify sample pretreatment. It does not require the traditional preconditioning, and thus the specific details about washing, suction, and elution solvent that you have asked for do not apply in this case. This feature is one of the primary reasons we selected this particular cartridge, as it streamlines the preparation process while maintaining a high degree of accuracy and precision. We have clearly specified "Oasis PRiME HLB Plus Light" in the manuscript to distinguish it from the traditional Oasis PRiME HLB. Zhang et al. (2018) have used HLB without any precondition, and we have described in Lines 198-199 that “According to the manufacturer's instructions and methods outlined in a previous paper [25], the organic supernatant (2 mL) was loaded into a syringe connected to the Oasis PRiME HLB plus light and passed through the cartridge.

R-Figure 1: Oasis PRiME HLB Plus Light cartridge

d. I acknowledge the lack of detail in the materials and methods (MM) section concerning the use of cartridge and alumina in sample preparation. We described dual purification methods in Lines 186-189, that “In addition, a dual purification was conducted on the extract obtained from the No. (8) procedure by further implementing the No. (2) or (7) purification methods. The purification efficiency of each preparation method was compared in three types of herbal medicines, taking into account both recovery rates and matrix effects”. To increase the removal efficiencies of across a wide ranges of interference sample matrices, dual purifications were considered. This strategy enables the elimination of impurities that a single purification method cannot remove, by employing an additional purification precedure. Gong et al. (2020) demonstrated that the combining dSPE and Oasis PRiME HLB resulted in superior matrix removal, compared to using HLB alone. In this study, we implemented a dual purification strategy by conducting an Oasis PRiME HLB plus light cleanup, followed by d-SPE containing C18 or Alumina sorbents. We described the statements in Lines 335-340.

e. Our skilled researchers took precautions to avoid introducing solid impurities when transferring extracts for GC-MS/MS analysis from dSPE, so filtration step was not required. In multiresidue study, some of pesticides may be caught by filter.

f. 10 ug pesticide/kg (ppb) in 5 g sample was extracted in 10 mL of extraction solvent, so the concentration became 5 ug pesticide/L extract solution. Without concentration or dilution of solution, the extract was subjected to matrix-matched (extract/solvent, 1:1, v/v), so concentration of pesticide in the final extract solution became 2.5 ug/L extraction solution. We added the following sentence to Lines 204-205 to clarify the correlation between the sample and the final extract: "The sample was equivalent to 0.25 g per 1 mL in the final extract."

g. Before extracting pesticides from the samples using ACN/EA, we thoroughly saturated the samples with distilled water to ensure that the pesticides within the sample particles were adequately extracted. To clarify the expression, we have modified "added" in Line 191 to "saturated.": The homogenized sample (5 g) in a 50-mL centrifuge tube was added saturated with 10 mL of distilled water for 30 min to ensure sufficient soaking. In addition, the water residue was isolated using liquid-liquid partitioning to capture polar compounds in aqueous layer. This crucial detail was not explicitly stated in our original manuscript, and we understand that this might have led to some confusion. We have now included this information in Lines 196-198.

h. According to the editor’s valuable comments, we have added the following sentences in Lines 174-176 (In the overall extraction studies, we compared the extraction patterns across three types of herbal medicines: *C. officinale, R. glutinosa, and P. lactiflora*) and Lines 187-189 (The purification efficiency of each preparation method was compared in three types of herbal medicines, taking into account both recovery rates and matrix effects)

i. In response to your inquiry about statistical methods, we used mean values and relative standard deviation in the interpretation of our research findings, without the use of advanced statistics. Consequently, specific mentions of statistical processing were not included.

3. Result:

a. At different time segments, pulsed injection pressure had different results? Why? No discussion is made.

b. Using EA, authors invited co-extracts as pointed out by the reviewers. Using water for saturation, this could have been avoided.

**Answer**: Thank you for your valuable comments regarding the Results section.

a. We understand the reviewer's concern about the different results at different time segments of pulsed injection pressure, which was not extensively discussed in our manuscript. Our team attempted to interpret the varying changes depending on the retention time. However, as we are not experts in the field of studying the mechanical movements of GC equipment, a clear interpretation of the flow dynamics and mechanical interactions is challenging. It is also notable that we couldn't find any relevant mention in other scientific papers that studied pulsed injection pressure. We acknowledge this as a limitation of our current study, and we are planning to conduct a follow-up research to elucidate this issue.

b. We used water in sufficient amounts to saturate the sample before extracting pesticides with organic solvents. The water used in this process was subsequently separated into water and organic solvent layers by performing liquid-liquid partitioning with QuEChERS salts. This procedure allowed us to confine polar co-extracts, such as sugars, in the water. This crucial detail was not explicitly stated in our original manuscript, and we understand that this might have led to some confusion. We have now included this information in Lines 196-199.

**Reviewer #3:**

1. Authors have specified LOQ as low as 0.002 ppm for most of the pesticides analysed. But the recovery tested were well above the respective LOQ values, which is a mandatory requirement for method validation. Authors have quantified the pesticides at above LOQ level but the recovery at LOQ level is lacking. The supporting information in form of tested results may be supplied for more accuracy of the method.

**Answer**: We greatly appreciate the reviewer's meticulous attention to our manuscript. we would like to clarify that the definition of LOQ is the minimum concentration at which a signal-to-noise ratio of 10 or more is satisfied in the chromatogram (Lines 209-210), and recovery tests are not separately required at this level. The LOQs of target pesticides in our study is a suggestion of the highest sensitivity that can be output in the developed analytical method. As the purpose of pesticide multiresidue analysis is to check rapidly whether non-registered pesticides exceed 0.01 mg/kg in crops. Therefore, it is crucial to have quantitation at this concentration. Therefore, it is typical in multiresidue analysis to perform recovery study with 0.01 mg/kg as the minimum concentration.

2. The choice of solvents for extraction in a ratio has been already addressed by previous reviewers. The authors have tried to answer, but I have a doubt about the solubility of the pesticides in the mixture. Can the authors discuss about this.

**Answer**: We are grateful for the reviewer's thoughtful question. The choice of solvents, acetonitrile (ACN) and ethyl acetate (EA), was not arbitrary. Both ACN and EA are organic solvents with high solubility for the pesticides. The solvents were selected based on their polarity and ability to extract a wide range of pesticide residues with varying polarities. In our preliminary tests, we confirmed that these solvents could efficiently solubilize all the pesticides we tested, leading to satisfactory recovery rates. We have included this additional detail in Lines (243-245) to provide a more comprehensive explanation of our solvent choice and ensure the scientific rigor of our method is accurately portrayed.

3. As regards to the clean up steps, the authors have utilised alumina to remove the co-extractives. But, the authors have addressed the reviewers question, stating that ethyl acetate results in more extraction of co-extractives, as observed in increased dry weight. This creates ambiguity. The authors have first increased the co-extractives through extraction and then they are trying to remove the co-extractives using alumina. This requires justification.

**Answer**: Thank you for your valuable comments. We understand the seeming contradiction the reviewer points out in terms of increasing co-extractives through extraction, and then trying to remove them using alumina. However, this process is essential to our approach and allows us to optimize multiresidual pesticide recoveries. In our methodology, we added ethyl acetate to the acetonitrile (ACN) extraction solvent, which we found to improve the recovery of certain pesticides that were not adequately extracted with ACN alone. While beneficial for pesticide recovery, indeed increased the extraction of co-extractives. To counteract this, we employed a dual purification process, effectively minimizing pesticide loss and maximizing purification efficiency. We understand that this step may seem paradoxical, but it enables a high recovery of pesticides that could not be achieved with the sole extraction by ACN. We sought to resolve any misunderstandings by explaining the principles of the established analytical method in Lines 351-356 before describing the validation of the method.

4. TIC of control samples are presented but they should have also presented the TIC of fortified matrix.

**Answer**: We understand the reviewer's suggestion to present the Total Ion Chromatogram (TIC) of the fortified matrix. However, the fortified matrix includes peaks from all 296 target pesticides, which makes it extremely difficult to distinguish individual peaks in one TIC. We have confirmed that the peaks of the 296 pesticides are distinctly distinguishable from the baseline in the three herbal matrices.
